# Nanoliposomes Permeability in a Microfluidic Drug Delivery Platform across a 3D Hydrogel

**DOI:** 10.3390/pharmaceutics16060765

**Published:** 2024-06-04

**Authors:** Corentin Peyret, Aleka Manousaki, Sabine Bouguet-Bonnet, Emmanuel Stratakis, Laura Sanchez-Gonzalez, Cyril J.F. Kahn, Elmira Arab-Tehrany

**Affiliations:** 1 Université de Lorraine, LIBio, F-54000 Nancy, France; corentin.peyret@univ-lorraine.fr (C.P.); laura.sanchez-gonzalez@univ-lorraine.fr (L.S.-G.); cyril.kahn@univ-lorraine.fr (C.J.F.K.); 2 Institute of Electronic Structure and Laser (IESL), Foundation for Research and Technology Hellas (FORTH), 711 10 Heraklion, Greece; manousa@iesl.forth.gr (A.M.); stratak@iesl.forth.gr (E.S.); 3 Université de Lorraine, CNRS, CRM2, F-54000 Nancy, France; sabine.bonnet@univ-lorraine.fr

**Keywords:** microfluidic, membrane barrier, gelatin methacryloyl, nanoliposomes, apparent permeability, porosity

## Abstract

Nanoliposomes are nano-sized vesicles that can be used as drug delivery carriers with the ability to encapsulate both hydrophobic and hydrophilic compounds. Moreover, their lipid compositions facilitate their internalization by cells. However, the interaction between nanoliposomes and the membrane barrier of the human body is not well-known. If cellular tests and animal testing offer a solution, their lack of physiological relevance and ethical concerns make them unsuitable to properly mimic human body complexity. Microfluidics, which allows the environment of the human body to be imitated in a controlled way, can fulfil this role. However, existing models are missing the presence of something that would mimic a basal membrane, often consisting of a simple cell layer on a polymer membrane. In this study, we investigated the diffusion of nanoliposomes in a microfluidic system and found the optimal parameters to maximize their diffusion. Then, we incorporated a custom made GelMA with a controlled degree of substitution and studied the passage of fluorescently labeled nanoliposomes through this barrier. Our results show that highly substituted GelMA was more porous than lower substitution GelMA. Overall, our work lays the foundation for the incorporation of a hydrogel mimicking a basal membrane on a drug delivery microfluidic platform.

## 1. Introduction

Nanoliposomes are considered as the most successful drug delivery system, with 15 liposomal drugs approved for clinical uses [1,2]. With a diameter of between 100 and 200 nm, their unique structure confers them the ability to encapsulate both hydrophilic and hydrophobic compounds [3,4,5]. The enhanced permeability and retention (EPR) effect helps nanoliposomes easily deliver their payload intracellularly. For this reason, they have garnered significant attention in recent years in various biomedical applications such as drug delivery and cancer therapy [6,7,8,9].

An important aspect of their utilization lies in comprehending their diffusion pathways through epithelial membranes via the trans- and para-cellular transporter pathway [10,11]. To study the interaction between nanoliposomes and epithelial membranes, there is a wide variety of tools to choose from animal models to Petri dish culture and Transwell^®^ systems [12,13,14,15,16]. However, while cell cultures have a high throughput, they lack physiological relevance and if animal models are more relevant to mimic the in vivo mechanisms, there is an imperative to reduce our reliance on animal experimentation for ethical reasons [17,18,19]. Finally, testing the products from a laboratory scale to clinical trial is a slow process and there is a growing demand for in vitro tools and methodologies that mimic human epithelial tissues. The Transwell^®^ system is a widely used technique that consists of two superimposed compartments that rely on passive particle diffusion. While they can mimic somewhat accurately the diffusion coefficient of drugs compared to in vivo values, their simplified structure, and the lack of active flow rate for compound diffusion, does not thoroughly recapitulate in vivo conditions.

In the last decade, microfluidic systems, more specifically, organ-on-a-chip, have been proposed to address this issue. Organ-on-a-chip microfluidic devices usually consist of two parallel channels representing either side of the epithelium and are separated by a membrane on which cells are cultured [20,21]. Microfluidic devices allow us to mimic the circulation of fluid to mimic blood flow and mechanical contraction of the membrane for a more accurate imitation of the in vivo environment. The addition of mechanical stimuli has been demonstrated to influence cell fate and to help recreate tissue-like structures observed in vivo (e.g., villi for gut) [22,23]. Such models offer a means to probe nanoliposome interactions with epithelial barriers in a controlled and physiologically relevant environment. However, these microfluidic models consist of a single cell layer on top of a polymer membrane, and they lack the presence of a basal membrane for the cells. The latter has been shown to direct cell fate, and its presence could therefore play a role to better mimic biological structures such as epitheliums [24,25]. Therefore, the addition of a hydrogel mimicking the basal membrane would be a step forward in the re-creation of a more physiologically relevant environment, and studying the passage of nanoparticles through them would help in understanding the interaction between nanoliposomes and epitheliums.

Here, we looked at the diffusion of nanoliposomes across a membrane in a microfluidic system. First, we determined the optimum parameters for promoting the passage of nanoliposomes through the system’s membrane. Then, using these parameters, we added a hydrogel membrane made of gelatin methacryloyl and studied the diffusion of nanoliposomes through it, where we found that the addition of the hydrogel did not obstruct the passage of nanoliposomes. This work is the first step toward establishing a more complex system, with the next being the addition of cells on the hydrogel to mimic an epithelium.

## 2. Materials and Methods

### 2.1. Reagents

Gelatin from porcine skin (Type A, 300 bloom), methacrylic anhydride (MA), Irgacure 2959 (PI) (2-hydroxy-4′-(2-hydroxyethoxy)-2-methylpropiophenone), Dulbecco’s phosphate-buffered saline (DPBS), and hexamethyldisilazane (HMDS) were purchased from Sigma-Aldrich (Saint-Quentin-Fallavier, France). 1,1′-Dioctadecyl-3,3,3′,3′tetramethylindocarbocyanine-perchlorate (Dil stain) was purchased from Thermo Fisher, France (Illkirch-Graffenstaden, France). Deuterium oxide (99.9 atom% D) was purchased from Eurisotop (Saint-Aubin, France).

### 2.2. GelMA Synthesis

Two different GelMA were synthesized as previously described [26]. Synthesis conditions were adjusted to obtain two different degrees of substitution. Briefly, gelatin was dissolved at 10% in DPBS at 50 °C under stirring. Two different ratios of methacrylic anhydride were added at a flow rate of 2 mL/min, at a 27.5% and 102.5% ratio compared to gelatin (*v*/*w*), respectively, with a syringe pump (KD scientific, Holliston, MA, USA). The mixture was let to react under an agitation of 650 rpm, for 2 h at 50 and 55 °C, respectively, for both samples. These values were chosen according to our previous work [27]. Three times the reaction volume of warm (50 °C) DPBS was added after 2 h to stop the reaction. Then, to get rid of excess salt and unreacted methacrylic anhydride, the mixture was dialyzed with 12–14 kDa cut-off dialysis tubing (SERVA Electrophoresis, Heidelberg, Germany) at 50 °C for 5 days against distilled water. For longer conservation, freeze-drying was used on GelMA for at least 48 h. The resulting foam was recovered and frozen at −20 °C for later usage.

### 2.3. Degree of Substitution Calculation

The degree of substitution (DoS) for GelMA was determined by proton nuclear magnetic resonance. A Bruker Avance III 400 MHz instrument (Bruker, Karlsruhe, Germany) was used at 40 °C and 400 MHz (^1^H resonance frequency) to collect the spectra. GelMA was dissolved at 50 °C in 1 mL of deuterium oxide at 50 °C. Quantitative measurements were acquired with 64 accumulations and a relaxation delay of 7 s, pre-saturation was used to eliminate the residual water signal. Peak areas for the region of interest were measured on phased and baseline corrected NMR spectra. The following equation was used to calculate the DoS:

(1) $$DoS=\left(1-\frac{Area\;lysine\left[GelMA\right]}{Area\;lysine\left[Gelatin\right]}\right)*100$$

### 2.4. Porosity

#### 2.4.1. Sample Preparation

Crosslinked GelMA was dehydrated by being placed in a water–ethanol washing bath for 7 min starting at 30% ethanol, up to absolute ethanol with a 10% increase step. Next, samples were placed in a mix with a ratio of 2:1, 1:1, and 1:2 ethanol and hexamethyldisilazane (HDMS) for 15, 5, and 5 min, respectively. Samples were rinsed three times with pure HDMS for 5 min. Finally, the HDMS was allowed to evaporate. Then, the dried hydrogels were sputter coated using a Bal-Tec SCD 050 (BAL-TEC AG, Balzers, Liechtenstein) with a 10 nm Au layer before the field emission scanning electron microscopy analysis.

#### 2.4.2. Scanning Electron Microscopy (SEM)

Field emission scanning electron microscopy was performed using a JEOL 7000 (JEOL Ltd., Tokyo, Japan) field emission scanning electron microscope with an acceleration voltage of 20 kV at various magnifications.

#### 2.4.3. Image Analysis

FEMS images were analyzed using Fiji software (ImageJ 1.54f), and the pore size was measured on scaled images using the measuring tool, using the pores’ largest section [28]. Four representative images of each gel were analyzed, and at least 100 pores were measured in each image.

### 2.5. Nanoliposomes

#### 2.5.1. Synthesis

For the formulation of nanoliposomes, 200 mg of rapeseed lecithin and 4 mg of Dil (1,1′-dioctadecyl-3,3,3′,3′-tetramethylindocarbocyanine perchlorate) for Dil nanoliposomes were dissolved in 9.8 and 9.796 mL of distilled water, respectively. The suspension was then mixed overnight under constant stirring in an inert atmosphere (nitrogen) and sonicated at 40 kHz and 40% of full power for 240 s (1 s on and 1 s off cycles) using a probe sonicator (Vibra-Cell™ VC 75115, Sonics & Materials Inc., Newtown, CT, USA) to obtain a homogeneous solution of nanoliposomes.

#### 2.5.2. Physico-Chemical Characterization

The average hydrodynamic particle diameter (Hd), polydispersity index (PDI), and ζ-potential of the prepared nanoliposomes were characterized by diffusion light scattering (DLS) with Zetasizer Nano ZS equipment (Malvern Instruments Ltd., Malvern, UK). Prior to measuring the size and ζ-potential, the samples were diluted (1:200) with ultrapure distilled water. Measurements were performed at 25 °C with a fixed scattering angle of 173°; the refractive index (RI) was set at 1.471 and the absorbance at 0.01. Size measurements were performed with a disposable cell, 1 cm in length (DTS 0012). Zeta potential measurements were performed in standard capillary electrophoresis cells equipped with gold electrodes (DTS 1070). At least three independent measurements were performed for each condition.

### 2.6. Microfluidic Chip

The Micronit organ-on-chip microfluidic device (Micronit, Enschede, The Netherlands) consisted of three 15 × 45 mm glass slides that formed two microfluidic chambers 250 µm in height. These two chambers were separated by a 4.02 cm^2^ PET membrane with 8 µm pores at a density of 6000 pores/cm^2^. The height of the chip portion above the porous membrane was 650 µm and the membrane itself was 160 µm thick. The top channel volume was equal to 110 mm^3^ and the bottom channel had a volume of 75 mm^3^.

### 2.7. Casting of GelMA

GelMA was diluted in warm (50 °C) PBS at 10% (*w*/*v*) with the addition of 0.5% (*w*/*v*) Irgacure 2959 photo-initiator. GelMA was cast onto the chip membrane following this procedure: 100 µL of warm GelMA was added on the chip membrane and spread evenly so that GelMA covered the whole area, then 40 µL was taken off to leave 60 µL on the membrane. GelMA was crosslinked using a CL-1000L ultraviolet crosslinker (UVP, Cambridge, UK) for 4 min. Thereafter, crosslinked GelMA was placed in 37 °C PBS for at least 3 h so that the quantity of water that was present inside the gel balanced itself with PBS.

### 2.8. Microfluidic System Operation

When in use, the chip was placed in a custom-made chip holder that locked all parts in position, and special ferules allowed for the connection of external tubing. The tubes used were made from polyetheretherketone (PEEK) with a 250 µm inner diameter. Two tubes, 20 cm long, were connected to either inlet for the top and bottom channel. Dil nanoliposomes, at a concentration of 50 µg of lecithin/mL, were flowed through the microfluidic device in the top channel at different flow rates, and PBS was flowed in the bottom channel at different flow rates, as detailed in Table 1, with two syringe pumps (KD scientific, Holliston, MA, USA). For experiments with gel cast onto the PET membrane, the flow rate ratio used was 2. Two tubes, 15 cm in length, were connected in both outlets, and were poured into two 15 mL collection tubes, one for the top and bottom channel, respectively. Collectors’ tubes were changed every hour, for four hours, and their contents were later analyzed by UV–Vis spectrophotometry. All experiments were carried out at least three times.

### 2.9. UV–Vis Spectrophotometry

Samples were collected from both outlets and their absorption at 550 nm was measured using a Shimadzu 1280 UV–Vis spectrophotometer (Shimadzu Ltd., Kyoto, Japan). Concentrations of Dil nanoliposomes was calculated using Beer–Lambert’s law (Equation (2)) and a standard curve. The standard curve was established by diluting a concentrated solution of nanoliposomes with a known concentration into six points: 100, 75, 50, 25, 10, and 5 µg of lecithin/mL.

(2) Abs=ε∗l∗C

where ε is the mass absorption coefficient of Dil, l is the length of the cuvette (1 cm), and C is the concentration in µg of lecithin/mL.

### 2.10. Apparent Permeability Coefficient Calculation

The apparent permeability coefficient (P_app_ expressed in cm/s) was calculated according to Equation (3).

(3) $$P_{app}=\frac{dC}{dt}*\frac{1}{A}*\frac{Q}{C_{0}}$$

where C is the measured concentration at the bottom outlet, in µg/mL, A is the exchange surface area (4.02 cm^2^), Q is the top channel inlet flow rate in cm^3^/s, and C_0_ is the inlet concentration (50 µg/mL). Results are depicted as the mean value ± standard deviation of three experiments.

## 3. Results and Discussion

### 3.1. GelMA Degree of Substitution

**Figure 1:**
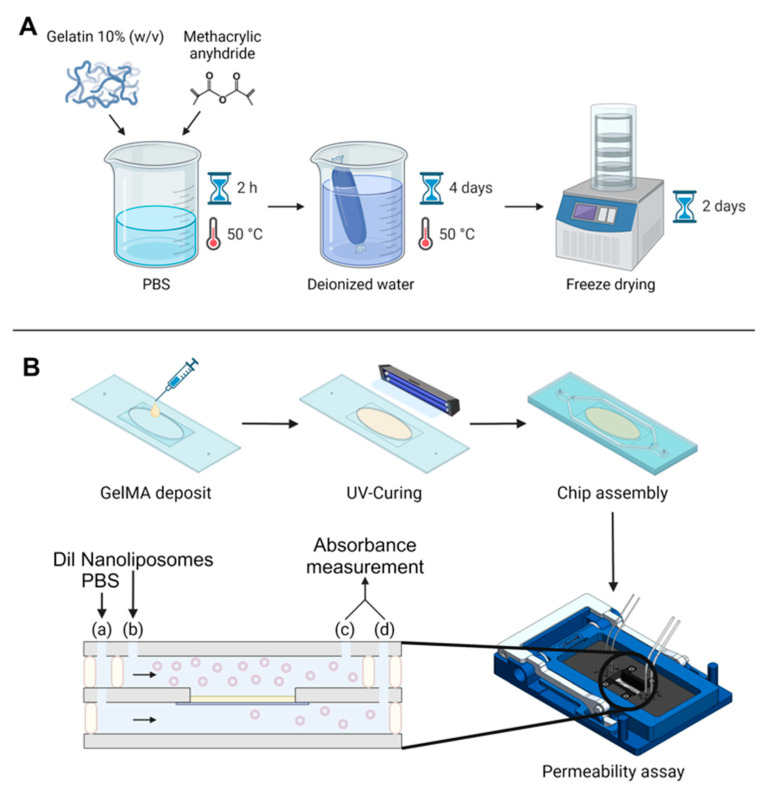
Global experimental pattern. (**A**) Synthesis protocol for GelMA. (**B**) Protocol for GelMA integration in the chip, chip assembly, and permeability assay. The black arrows indicate flow direction, beige rectangle symbolizes the GelMA membrane. Two syringe pumps were used at two different flow rates; fluids were injected in the microfluidic chip with a flow rate ratio of 2: (a) 30 µL/min for Dil nanoliposomes in PBS and (b) 15 µL/min for PBS. Chip outputs (c) and (d) were collected from both the bottom and top channel every hour for absorbance measurement by UV–Vis spectroscopy.

GelMA was synthesized using two different synthesis conditions (Figure 1A), and the degree of substitution was characterized with ^1^H NMR.

**Figure 2:**
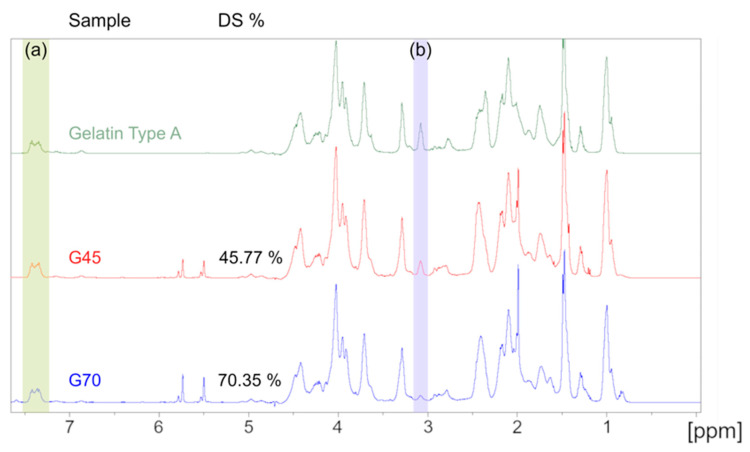
^1^H NMR spectra of the unmodified gelatin, G45, and G70, respectively. (**a**) 7.23–7.5 ppm: aromatic amino acid peak used as reference. (**b**) 3–3.2 ppm: lysine methylene protons’ peak. The area under these peaks were used to calculate the degree of substitution.

Due to its complex amino acid structure, gelatin and GelMA have a complex spectrum when analyzed with ^1^H NMR. Therefore, lysine substitution rate quantification was calculated by using the peak area of the aromatic amino acid region (d = 7.23–7.5 ppm) of gelatin as a reference between the different samples. The lysine methylene protons’ peak area located between d = 3 and 3.2 ppm was compared between gelatin and the two samples to calculate the DoS. The resulting spectra are presented in Figure 2 and confirmed the substitution of the amine group on the lysine amino acid. A degree of substitution of 45.77 ± 0.49% and 70.35 ± 0.84% was calculated for either gel, thus, they are referred to as G45 and G70, respectively, thereafter. These values are in accordance with the experimental design that was previously conducted, validating the model we established as well as the previously reported degree of substitution [29].

### 3.2. GelMA Microstructure

**Figure 3:**
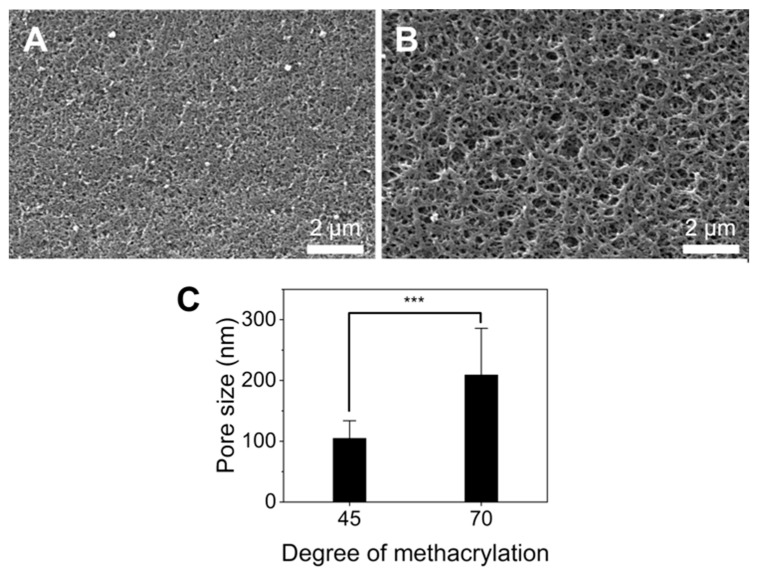
Porosity of GelMA. (**A**,**B**) Representative SEM images of the G45 and G70 surfaces. Scale bar: 2 µm, respectively. (**C**) Pore size for G45 and G70, average of n = 460 *** = relevance > 99.99% (for *p* value < 0.001).

Hydrogel porosity is important for the diffusion of molecules and nanoparticles through their structure [30]. Thus, the GelMA microstructure was studied using SEM images to better understand the diffusion of nanoliposomes in the gel. The results are presented in Figure 3. SEM images revealed that G70 was more porous than G45, with an average pore size of 210.4 ± 90.4 nm and 106.6 ± 34.2 nm, respectively (Figure 3C). It has been shown in previous studies that GelMA with a higher degree of substitution generally has smaller pores [31,32]. Many parameters are known to influence the pore size of crosslinked GelMA such as the gel crosslinking time and technique as different photo-initiators and crosslinking times change GelMA’s physical properties and porosity [33,34]. Notably, longer crosslinking times led to lower pore sizes; GelMA was UV-cured for 4 min in this study, which is in the upper range of curing times [35,36]. Additionally, our 221 mean sub-micrometric pore size was significantly lower than the one usually reported for GelMA, which is in the micrometer range [36,37]. A recent study comparing freeze-drying and solvent drying on GelMA microgels mixed with hyaluronic acid and collagen showed that freeze-dried hydrogels had much bigger pores (31–49 µm) than the isopropanol-dried one (0.5 µm), therefore the discrepancy between our results and other studies could be due to the drying method, as freeze-drying is usually preferred [38]. The shrinkage of the sample during the dehydration process suggests that the pore size in a hydrated sample should be somewhere between the pore size measured by freeze-drying and solvent drying. Therefore, the combination of both crosslinking conditions with a solvent dehydrating method could be responsible for the different pore sizes observed compared to the literature.

### 3.3. Nanoliposomes

**Figure 4:**
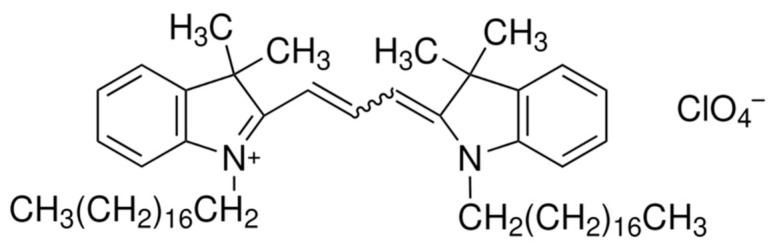
Chemical structure of Dil.

To quantify the concentration of nanoliposomes, a dye was used to measure their concentration, as blank nanoliposomes do not absorb or emit any light. Dil dye was chosen to stain the nanoliposomes. Indeed, long aliphatic chains enable Dil to intercalate into the liposome membrane; its structure is displayed in Figure 4. Moreover, Dil is especially interesting, because, unlike many fluorescent products, it has been demonstrated that Dil does not leak out of the nanoliposome membrane to the outside medium and is stable over a longer period of time [39].

**Figure 5:**
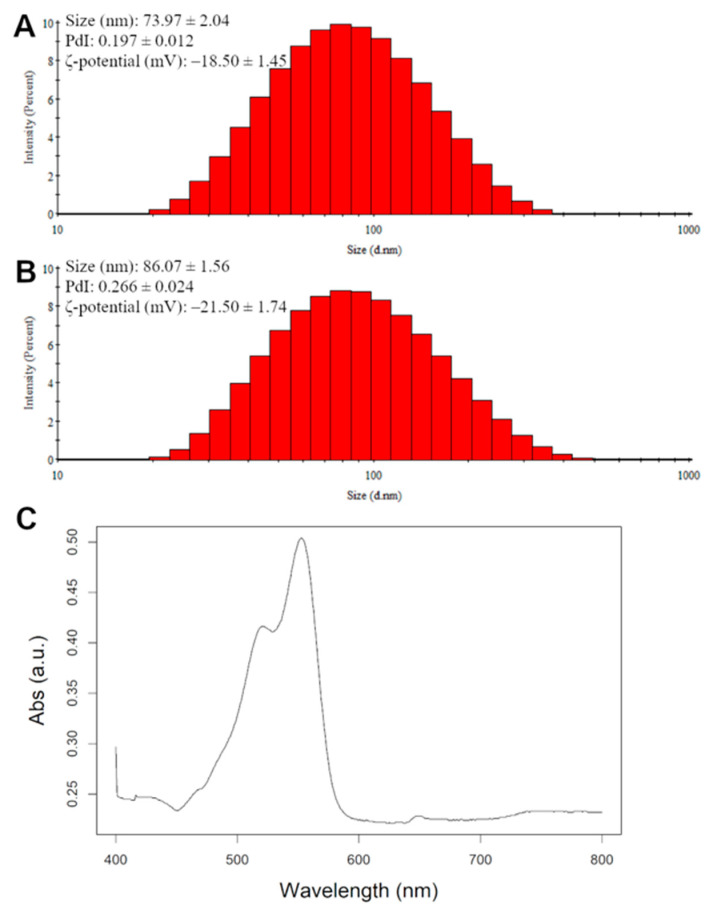
Physico-chemical characterization via DLS of nanoliposomes (**A**) and Dil nanoliposomes (**B**). (**C**) absorption spectra of Dil-loaded nanoliposomes.

The nanoliposomes and Dil nanoliposomes had a mean Hd of 74 nm and 86 nm, with a PDI of 0.197 and 0.266 for the nanoliposomes and Dil nanoliposomes, respectively (Figure 5A,B). Dil nanoliposomes were 14% bigger than their pure rapeseed lecithin counterparts and their PDI was higher, suggesting a less monodisperse solution probably due to the inclusion of Dil in the phospholipid bilayer (Table 2). The PDI value remained below 0.3, which is considered to be acceptable for monomodal nanoliposomal formulations [40]. These data indicate that the addition of Dil increased the size of the nanoliposomes by a small margin, but the nanoliposomes were still smaller than the average pore size for each sample.

Zeta potential was used to measure the surface charge of the nanoparticles, where a positive or negative value means that the nanoparticles are more stable in a solution since they would repel each other. The ζ-potential for nanoliposomes is found to be generally negative due to the presence of phospholipids in their composition [41]. In our study, the nanoliposomes showed a slightly lower surface charge (−18.50 mV) compared to the Dil nanoliposomes (−21.50 mV); a cause could be the higher Hd of the nanoliposomes (Table 2) causing more phospholipids to be exposed on their surface as well as the presence of perchlorate ions. Absorbance spectra of the Dil nanoliposomes were measured with a UV–Vis spectrophotometer. The spectrum of absorption against wavelength is shown in Figure 5C, where the maximum absorption wavelength was measured to be 550 nm, therefore, all measurements were carried out at that wavelength.

### 3.4. Nanoliposomes Diffusion across GelMA Hydrogel

As a first step, the microfluidic system displayed in Figure 1B was calibrated and studied to check whether the PET membrane was an obstacle to nanoliposome diffusion in comparison with a chip without membrane (Table 3). It was shown in the flow simulation that, in this chip, because its dimensions are in the micrometer scale, the liquid is almost still near the membrane [42]. Consequently, different flow rates were tested to induce a pressure difference between the top channel, where the liposomes are injected, and the bottom channel, where the liposomes are collected (Table 1).

It is apparent from Table 3 that the apparent permeability coefficient stabilized after 1 h. Moreover, the flow rate ratio significantly impacted the permeability coefficient value. From ratio 1 to 1.25, a 2.8-fold increase was observed and from ratio 1 to 2, there was a 6.7-fold augmentation for the membrane free chip. Whereas for the chip with a membrane, the permeability coefficient showed a 3.0- and 7.5-fold increase, respectively. Statistical analysis, 3-way ANOVA with a post-hoc student test, did not show a significant difference at the 0.05 level between P_app_ in the chip with and without the membrane. Consequently, the membrane was not an obstacle to the passage of nanoliposomes, as the coefficient dropped from an average of (3.84 ± 0.03) × 10^−5^ to (3.40 ± 0.23) × 10^−5^ cm/s at a flow rate ratio of 2. Moreover, the data from different ratios showed a statistically significant difference, confirming the fact that higher ratios have higher P_app_. Therefore, for experiments with a GelMA membrane, the flow rate ratio was fixed at 2.

In a second step, GelMA was cast atop the PET membrane and nanoliposomes were flowed into the chip with a flow rate ratio of 2. The P_app_ coefficient of nanoliposomes through the hydrogel membrane was measured; this experiment was performed twice, and the results are presented in Supplementary Table S1. From the table, P_app_ diminished significantly, by an order of magnitude, compared to the values obtained in a chip with a PET membrane only.

At a flow rate ratio of 2, the P_app_ coefficient over the course of 4 h went from an average of (3.40 ± 0.23) × 10^−5^ cm/s to (3.24 ± 0.17) × 10^−6^ cm/s and (6.33 ± 1.61) × 10^−6^ cm/s for the chip with a PET membrane, G45, and G70, respectively. These results indicate that the addition of GelMA impaired the passage of nanoliposomes when compared to the chip with the PET membrane. Moreover, the P_app_ coefficient was overall higher for G70, with a value between 15.13 and 7.83 × 10^−6^, 13.45 and 10.44 × 10^−6^, and 6.72 and 7.83 × 10^−6^ cm/s compared to G45, which had a value comprised between 2.53 and 4.17 × 10^−6^, 3.11 and 3.57 × 10^−6^, and 3.11 and 2.98 × 10^−6^ cm/s for 2, 3, and 4 h, respectively (Supplementary Table S1). Values for the first hour were not considered because it corresponded to the time required for the system to reach equilibrium. These results were consistent with the previous observation that the mean pore size was higher for G70 than G45: a higher pore size translated into a higher P_app_ coefficient. P_app_ values for G45 were in the same order of magnitude as one previously reported for simulated intestinal epithelium with CaCo-2 cells in Transwell^®^ systems, and for G70, the P_app_ values resembled the ones reported for a model of BBB in a Transwell^®^ system of around 8.0 × 10^−6^ cm/s between 1 and 3 h, going all the way to 16 cm/s after 4 h [11,43]. Moreover, the apparent permeability coefficients of the nanoliposomes through different hydrogels were reported to be much higher, between 46 and 60 × 10^−6^ cm/s for collagen and between 10 and 35 × 10^−6^ cm/s for Matrigel, which is in line with the value we found for G70 [44].

Finally, the P_app_ values were in the same order of magnitude with the one observed in vivo for the diffusion of nanoparticles and molecules as well as the one observed in Transwell^®^ systems with cells on the membrane, supporting the choice of inducing a pressure difference between the two channels [45,46]. The pore size and diameter of the nanoparticles have been shown to be linked regarding the P_app_ coefficient, and in our case, the pore size was greater than the nanoliposome diameter (86.07 ± 1.56 nm), favoring their passage [47]. Additionally, particle diffusion has been shown to be charge asymmetric, with the surface charge of nanoparticles being one of the factors that enhances nanoparticle diffusion in a hydrogel. Our nanoliposomes were negatively charged (Figure 5A,B), and gelatin type A is neutrally charged at pH 7.4, which is the PBS base pH [48,49]. Therefore, it is likely that the surface charge of the nanoliposomes did not hinder their passage though the membrane. This is encouraging for our system as it would mean that adding cells on top of the gel would probably yield a similar P_app_, as epithelial cells tend to internalize passing particles in suspension and transport them to the basal side [50,51].

## 4. Conclusions

In this work, we first synthesized two GelMA hydrogels with known physical properties and degree of substitution. We then characterized their pore density using a HDMA drying method. Unexpectedly, we found that the higher degree of substitution GelMA had a higher mean pore size, and that the HMDA drying method showed a significantly smaller pore size when compared to freeze-drying. Second, we examined the permeability of the hydrogel to nanoliposomes on a reusable microfluidic device and optimized the flow rate ratio between the two compartments. As expected, we found that the higher the flow rate ratio, the higher the P_app_, and that the presence of the PET membrane did not constitute a significant obstacle to the diffusion of the nanoliposomes. Finally, using the best flow rate ratio, we studied the diffusion of nanoliposomes through a GelMA membrane in our microfluidic device. We found that the addition of a polymer membrane did not obstruct the diffusion of the nanoliposomes, and that the P_app_ was correlated with the pore size of the hydrogel. This work is, to the authors’ best knowledge, the first work to study the passage of nanoliposomes through GelMA and is a first step toward a simulated epithelium with the presence of a basal membrane, which is absent in other systems.

## Figures and Tables

**Table 1:** Different inlet flow rate ratio to induce a pressure difference between the two channels.

Flow Rate Ratio	Top Flow Rate	Bottom Flow Rate
	µL/min	µL/min
1	25	25
1.25	25	20
2	30	15

**Table 2:** Nanoliposomes and Dil nanoliposomes: mean size, PDI, and ζ-potential.

	Nanoliposomes	Dil Nanoliposomes
Size (nm)	73.97 ± 2.04	86.07 ± 1.56
PDI	0.197 ± 0.012	0.266 ± 0.024
ζ-potential (mV)	−18.50 ± 1.45	−21.50 ± 1.74

**Table 3:** P_app_ measured at different flow rate ratios (1, 1.25, 2) after 1, 2, 3, and 4 h on the microfluidic setup with or without a membrane on the chip. Lines with the letter ^a^ showed a significant difference at the 0.05 level.

	P_app_ (10^−6^ cm/s)					
Ratio	1 (25/25) ^a^		1.25 (25/20) ^a^		2 (30/15) ^a^	
Time	w/Membrane	w/o Membrane	w/Membrane	w/o Membrane	w/Membrane	w/o Membrane
1 h	8.37 ± 5.58	8.43 ± 5.59	11.34 ± 4.14	22.02 ± 7.81	30.78 ± 5.47	38.20 ± 6.64
2 h	4.50 ± 0.97	5.14 ± 2.10	13.59 ± 4.17	15.55 ± 1.22	34.15 ± 2.74	38.10 ± 3.21
3 h	4.53 ± 1.31	5.61 ± 0.61	14.70 ± 3.63	15.93 ± 1.80	35.54 ± 2.25	38.46 ± 8.36
4 h	4.38 ± 1.48	6.54 ± 2.75	12.09 ± 3.62	15.74 ± 1.81	31.13 ± 2.97	38.72 ± 10.20

## Data Availability

Data are contained within the article and Supplementary Materials.

## Supplementary Materials

The following supporting information can be downloaded at: https://www.mdpi.com/article/10.3390/pharmaceutics16060765/s1. Table S1: P_app_ of nanoliposomes through GelMA hydrogel with two different degrees of substitution, data from two experiments.
