# Supplementary material for: Nanoliposomes Permeability in a Microfluidic Drug Delivery Platform across a 3D Hydrogel
Source: Pharmaceutics. 2024 Jun 4;16(6):765. doi: 10.3390/pharmaceutics16060765 (PMC11207390; doi:10.3390/pharmaceutics16060765)
Supplement: Supplementary file 1 [file pharmaceutics-16-00765-s001.zip › pharmaceutics-3006017-supplementary.pdf]

Supplementary Table S1: P_{app} of nanoliposomes through GelMA hydrogel with two different degrees of substitution, data from two experiments.

	P_{app} (10^{-6} cm/s)			
Time	G45-1	G45-2	G70-1	G70-2
1h	10.12	4.17	20.17	9.57
2h	2.53	4.17	15.13	7.83
3h	3.11	3.57	13.45	10.44
4h	3.11	2.98	6.72	7.83
